# Integrating Sugar Metabolism With Transport: Elevation of Endogenous Cell Wall Invertase Activity Up-Regulates *SlHT2* and *SlSWEET12c* Expression for Early Fruit Development in Tomato

**DOI:** 10.3389/fgene.2020.592596

**Published:** 2020-10-19

**Authors:** Lei Ru, Yong He, Zhujun Zhu, John W. Patrick, Yong-Ling Ruan

**Affiliations:** ^1^The Key Laboratory for Quality Improvement of Agricultural Products of Zhejiang Province, College of Agriculture and Food Science, Zhejiang Agriculture and Forestry University, Hangzhou, China; ^2^School of Environmental and Life Sciences, Australia-China Research Centre for Crop Improvement, The University of Newcastle, Callaghan, NSW, Australia

**Keywords:** cell wall invertase, tomato fruit, SWEET, hexose transporter, sucrose transporter, sugar metabolism

## Abstract

Early fruit development is critical for determining crop yield. Cell wall invertase (CWIN) and sugar transporters both play important roles in carbon allocation and plant development. However, there is little information about the relationship between CWIN and those functionally related sugar transporters during fruit development. By using transgenic tomato with an elevated CWIN activity, we investigated how an increase in CWIN activity may regulate the expression of sugar transporter genes during fruit development. Our analyses indicate that CWIN activity may be under tight regulation by multiple regulators, including two invertase inhibitors (INVINHs) and one defective CWIN (deCWIN) in tomato ovaries prior to anthesis. Among the sugar transporters, expression of *SlSWEET12c* for sucrose efflux and *SlHT2* for hexose uptake was enhanced by the elevated CWIN activity at 10 and 15 days after anthesis of tomato fruit development, respectively. The findings show that some specific sugars will eventually be exported transporters (SWEETs) and hexose transporters (HTs) respond to elevate CWIN activity probably to promote rapid fruit expansion when sucrose efflux from phloem and hexose uptake by parenchyma cell are in high demand. The analyses provide new leads for improving crop yield by manipulating CWIN-responsive sugar transporters, together with CWIN itself, to enhance fruit development and sugar accumulation.

## Introduction

Tomato is one of the most important fleshy fruit crops globally (Schwarz et al., 2014). Following pollination and fertilization, tomato fruit undergoes rapid cell division and expansion prior to maturation. Early development of tomato fruit is a critical period for determining fruit traits, such as fruit size and sugar accumulation (Mounet et al., 2009), hence the final fruit yield and quality (Ruan et al., 2012; Azzi et al., 2015).

Ovary and young fruits are typical carbon sinks, which require sugars transported from source leaves. In these sinks, sucrose can be unloaded into sink cells through either symplasmic or apoplasmic unloading or both simultaneously (Patrick, 1997; Patrick et al., 2001). Phloem unloading is often temporally and spatially dynamic to match developmental and physiological demands. For example, in tomato fruit, unloading shifts from an apoplasmic route in the ovary wall to a symplasmic pathway in the pericarp of young fruit (Jin et al., 2009; Palmer et al., 2015) before switching to an apoplasmic route in the pericarp at the onset of the rapid phase of hexose accumulation (Ruan and Patrick, 1995). In contrast, unloading appears to occur apoplasmically from fruit placenta to ovules or developing seeds (Jin et al., 2009; Palmer et al., 2015). Symplasmic unloading requires intercellular interconnection by functional plasmodesmata (Lalonde et al., 2003), while apoplasmic unloading typically involves the expression of plasma-membrane sugar transporters and cell wall invertase (CWIN) hydrolyzing sucrose into glucose and fructose (Slewinski, 2011; Ludewig and Flugge, 2013; Li et al., 2017). The sugar transporters include sucrose transporters (SUTs), hexose transporters (HTs), as well as sugars will eventually be exported transporters (SWEETs).

Cell wall invertase (CWIN) and sugar transporters have been studied extensively but separately. SlLin5 is the major CWIN expressed during tomato fruit development (Fridman and Zamir, 2003; Ruan, 2014; Palmer et al., 2015). SlLin5 has also been shown to locate in QTL sites that determine fruit weight (Fridman et al., 2004) and sugar content (Tieman et al., 2017). Silencing *SlLin5* resulted in fruit abortion (Zanor et al., 2009), while increasing CWIN activity sustained fruit set under heat stress by suppressing programmed cell death (Liu et al., 2016). Identification and expression of SUTs and HTs have been investigated in tomato (e.g., Reuscher et al., 2014). SUTs, the smallest family among the three sugar transporters, have been extensively reviewed (e.g., Kuhn and Grof, 2010; Ayre, 2011; Reinders et al., 2012). In tomato, there are three SUTs, namely, SlSUT1, SlSUT2, and SlSUT4 (Hackel et al., 2006). For the HT family, three SlHTs (SlHT1–3) have been experimentally analyzed, which revealed that their preferred substrate is hexose (McCurdy et al., 2010). Other newly identified HTs are yet to be experimentally characterized (Reuscher et al., 2014). The tomato SWEET family has been examined on a genome-wide scale, including a total of 31 putative SlSWEET genes in the tomato genome (Feng et al., 2015; Shammai et al., 2018; Ho et al., 2019). The SWEETs may facilitate bidirectional transport of glucose and fructose (clades I and II), sucrose (clade III) across plasma membranes, and of sugars across tonoplasts of vacuoles (clade VI) (Chen et al., 2015a).

Both CWIN and sugar transporters can control sucrose allocation (Ludewig and Flugge, 2013; Schroeder et al., 2013). It is widely accepted that co-expression of CWIN and HTs exists to support the development of many sink organs, such as developing barley grains (Weschke et al., 2003), tomato (Shen et al., 2019), and apple fruits (Wang et al., 2020). Recently, heterologous expression of *MdHT2* increased CWIN activity (Wang et al., 2020). Conversely, Liao et al. (2020) reported that silencing AtCWIN2 and AtCWIN4 genes reduced the expression level of many clade II SWEETs for hexoses and H^+^-coupled HTs during ovule formation in *Arabidopsis*. Similarly, Sosso et al. (2015) found that ZmSWEET4c, transferring CWIN-mediated hexoses for seed filling in maize, was downregulated in a CWIN mutant, while reciprocally the expression of CWIN was reduced in a SWEET mutant (Sosso et al., 2015).

Despite the progress, as outlined above, on understanding the expression relationship between CWIN and sugar transporter genes in wild type (WT) plants and mutants or transgenic lines, where one of the two players was suppressed or knocked out, there has been no report as how sugar transporters may respond to elevated endogenous CWIN activity during tomato fruit development (Ru et al., 2017). Answering this question is important as it will not only fill the knowledge gap in understanding sugar metabolism and transport but could also provide new avenues to improve crop yield directly (Ruan, 2014). Here, we address this question by using transgenic tomato in which an inhibitor gene against CWIN was silenced leading to elevated CWIN activity (Jin et al., 2009).

## Materials and Methods

### Plant Materials

Transgenic plants (*SlINVINH1*-RNAi), in which CWIN activity is elevated by silencing its inhibitor gene, *SlINVINH1*, were used (Jin et al., 2009). Tomato plants (*Solanum lycopersicum* XF-2) were raised in a glasshouse under natural light and exposed to day/night temperatures of 25/18°C for 14 and 10 h, respectively. Plants were grown in 25 cm diameter (8 L) pots filled with potting mix (one part coarse sand, one part perlite, and one part coir-peat), with one plant per pot. Standard Osmocote™/Osmocote high (K) potassium™ 1:1 slow release fertilizer (Scotts) was applied at a rate of 20 g per pot, supplemented with a weekly liquid fertilizer regime of Jurox Wuxal Liquid Foliar Nutrient Fertilizer™ at a diluted concentration of 4 ml.L^−1^. Pots were maintained at field capacity by being irrigated twice a day, each of 3-min duration, by an automated drip irrigation system.

Fruit age was determined by tagging flowers on the day of anthesis. Hand-pollination was applied on the day of anthesis to ensure every flower was pollinated. Ovaries at 2-days before anthesis (2 dba) and fruits at 2, 5, 10, and 15 days after anthesis (daa) were harvested as reproductive tissues. This sampling regime captured important tomato fruit development, such as fruit set (2 dba ovaries to 2 daa fruitlets), cell division (2 daa fruitlets to 10 daa fruits), and onset of cell expansion (15 daa fruits). Sink and source leaves were collected from expanding and expanded leaves of the same grown plants, while roots and shoots were harvested from 2-week old young seedlings. Samples were stored at −80°C immediately after harvest.

### RNA Extraction and cDNA Synthesis

RNA was extracted with RNeasy Plant Mini kit (QIAGEN) with 20–50 mg fresh weight tissue depending on the samples used. Thirty microliter of RNase-free H_2_O was added to dissolve RNA in the final step. RNA quality and concentration were measured with NanoDrop1000™ spectrophotometer (Thermo Scientific). For all samples, OD260/280 was between 1.8 and 2.4, which is an indicator for good RNA quality. One microgram of total RNA was treated with RQ1 RNase-Free DNase (Promega) to degrade residual genomic DNA. The DNase-treated RNA samples were then converted into cDNA by using oligo(dT)_20_ primer and SuperScript™III reverse transcriptase (Invitrogen life technology), according to the manufacturer’s instructions. The cDNA was diluted 1:20 for semi-quantitative real-time PCR (semi-qRT-PCR) and quantitative real-time PCR (qRT-PCR).

### Semi-qRT-PCR

Semi-qRT-PCR was performed with Taq DNA polymerase (New England Biolabs). A PCR reaction (10 μl) comprised 10 × ThermoPol reaction buffer (1 μl), 10 mM dNTP (0.2 μl), 10 mM forward/reverse primers (0.25 μl each), Taq DNA polymerase (0.05 μl), diluted cDNA (1 μl), and autoclaved water (7.5 μl). PCR program was as follows: initial denaturation at 95°C for 3 min, followed by 25 cycles of denaturation at 95°C for 30 s, annealing at 60°C for 30 s, extension at 68°C for 30 s, and a final extension at 68°C for 5 min, and products were held at 4–10°C. After that, 2 μl loading dye (Thermo Scientific) was added into PCR products, and then 10 μl products with loading dye were electrophoresed on 1.5% agarose gel with 1:1,000 GelRed (Biotium). Bands of cDNAs were visualized in GelDoc XR Imaging System (BIORAD), and band intensity was measured with Image J 1.45 s. The cDNA templates were adjusted to make sure that the band intensity of PCR products from different tissues were equalized before amplifying *SlEF1a*. After the equalization, the same amount of cDNA of different tissues was applied to amplify all the genes of interest. The gene specific primers are listed in Supplementary Table S1.

### Selection of Reference Genes for qRT-PCR

There are no universal reference genes for all experimental conditions, thus screening stable reference genes is necessary for different tissues and stages selected in our study. Eight tomato tissues from different organs and fruits at different developmental stages were investigated including four vegetative tissues of roots and shoots from 2-week old seedlings, sink and source leaves, as well as four reproductive tissues of ovaries at 2 dba and fruits at 2, 10, and 15 daa. Seven candidate reference genes were assessed in this study (Supplementary Table S2).

### Quantitative Real-Time-PCR

Quantitative real-time-PCR was conducted according to Ru et al. (2017). Briefly, the same cDNAs for semi-qRT-PCR were used in qRT-PCR analyses. qRT-PCR was performed using Platinum Taq DNA polymerase and SYBR Green fluorescent dye (Invitrogen) on Rotor-gene Q (QIAGEN). PCR program was as follows: initial denaturation at 95°C for 10 min, followed by 45 cycles of denaturation at 95°C for 20 s, annealing at 60°C for 20 s, and extension at 72°C for 20 s. A melt curve was performed in each run to verify the amplification of a specific product with 0.5°C increment from 60 to 90°C. Relative expression was calculated using the Pfaffl method (Pfaffl, 2001). See Supplementary Table S3 for the gene specific primers used for qRT-PCR.

## Results

### Multiple Potential CWIN Regulators Expressed at Ovary Stages, Including Two INVINHs and One Defective CWIN

**Figure 1:**
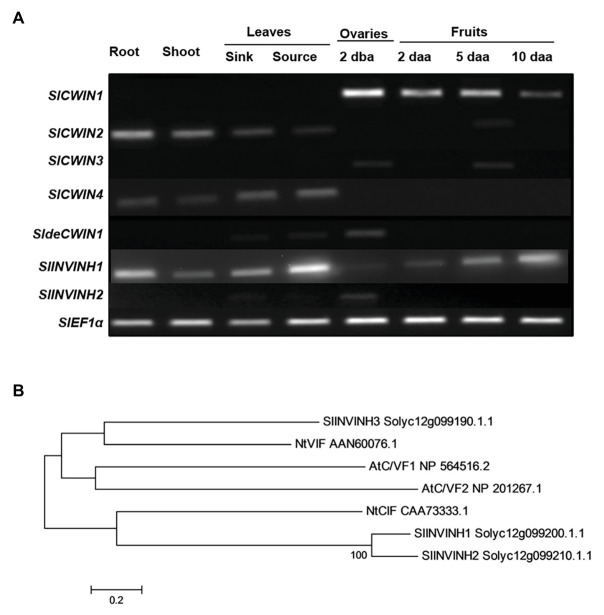
Cell wall invertase (CWIN) and its potential regulators. **(A)** Expression profile of CWIN (*SlCWIN1*–*SlCWIN4*), a defective CWIN (deCWIN; *SldeCWIN1*), CWIN inhibitors (*SlINVINH1* and *SlINVINH2*) in vegetative and reproductive tissues. Vegetative tissues included roots and shoots from 2-week old seedlings, sink, and source leaves from similar raised plants. Reproductive tissues included 2 days before anthesis (2 dba) ovaries and 2, 5, and 10 days after anthesis (daa) fruits. SlEF1α was used as reference gene. Original gel images in (A) are shown in Supplementary Figure S1. **(B)** Phylogenetic tree of invertase inhibitors. The tree was generated with MEGA 5.10 using the neighbor-joining method, following multiple sequences alignments with ClustalW. Species abbreviations: At, *Arabidopsis thaliana*; Nt, *Nicotiana tabacum*; and Sl, *Solanum lycopersicum*.

Cell wall invertase activity may be regulated by invertase inhibitors (INVINHs) and defective CWIN (deCWIN) together at the posttranslational level (Palmer et al., 2015). However, how CWIN activity is tightly regulated during early tomato fruit development is still unclear. To address this issue, the expression of four known CWINs (Fridman and Zamir, 2003), INVINHs (Qin et al., 2016), and deCWIN (Palmer et al., 2015) were investigated using RT-PCR. Among the four CWINs, the expression of *SlCWIN1*, previously named *SlLin5*, was only detected in reproductive tissues, not in vegetative tissues. Moreover, *SlCWIN1* is the main CWIN expressed during tomato fruit development, compared to SlCWIN2–SlCWIN4. Expression level of *SlCWIN1* decreased sharply in 2 daa fruitlets as compared to 2 dba ovaries and then was maintained at a relative stable level from 2 to 10 daa fruits (Figure 1A). The expression of a previously characterized CWIN inhibitor, *SlINVINH1* (Jin et al., 2009), is constitutively expressed in both vegetative and reproductive tissues (Figure 1A). However, *SlINVINH2*, a new CWIN inhibitor which clustered closely together with functional CWIN INVINH in tomato and NtCIF in tobacco (Figure 1B), was only expressed in the 2 dba ovary stages but not in vegetative tissues and other fruit stages (Figure 1A). *SldeCWIN1* is another potential regulator for regulating CWIN activity at post-translational level (Palmer et al., 2015) with its expression was only detected in 2 dba ovaries and sink leaves (Figure 1A). The findings indicate that CWIN activity could be regulated by two inhibitors, SlINVINH1 and SlINVINH2, and one defective invertase, SldeCWIN1, at the posttranslational level in tomato ovaries prior to fruit development.

### Elevated CWIN Activity Does Not Affect the Expression of SlSUTs

Firstly, Genorm was applied to obtain stable reference genes for qRT-PCR analyses (Vandesompele et al., 2002). The data were used to rank the reference genes expression in different tissues in Genorm (Supplementary Table S4). *SlCAC* and *SlSAND* were identified as the most stable reference genes for the qRT-PCR experiment (Supplementary Table S5).

**Figure 2:**
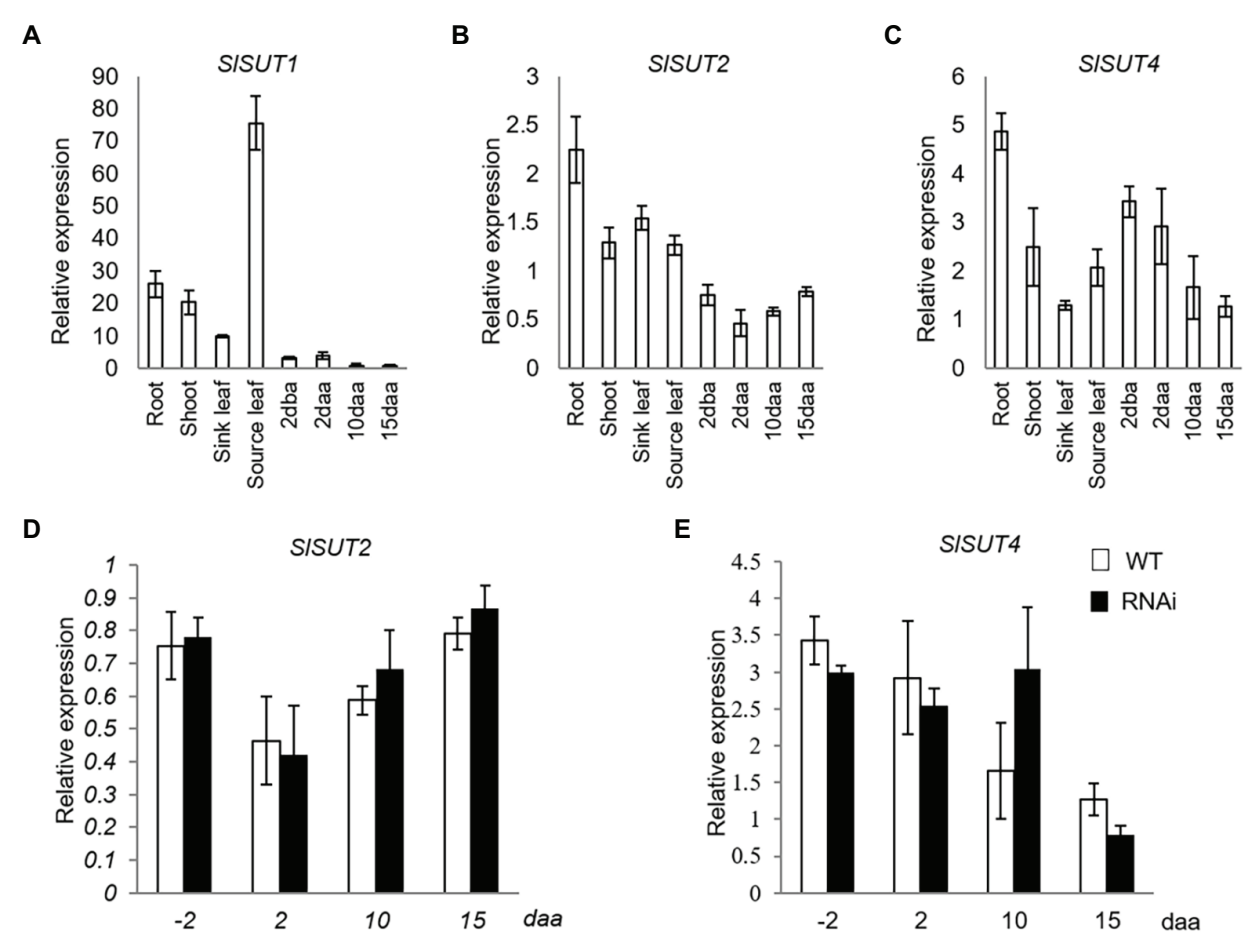
Quantitative real-time-PCR (qRT-PCR) analysis of the expression profiles of sucrose transporter (SUT) genes. **(A–C)** are expression profiles of SUTs in different tissues of tomato plants. **(A)** *SlSUT1*. **(B)** *SlSUT2*. **(C)** *SlSUT4*; **(D)** and **(E)** are expression profiles of SUTs in response to elevated CWIN activity in *SlINVINH1*-RNAi as compared to WT plants **(D)** *SlSUT2*. **(E)** *SlSUT4*. Tissues examined were vegetative and reproductive tissues. Vegetative tissues included root and shoots from 2-week old seedlings, sink, and source leaves from the similar-raised plants. Reproductive tissues included 2 dba ovaries and 2, 10, and 15 daa fruits. *SlCAC* and *SlSAND* were used as reference genes. Each value is the mean ± SE of three biological replicates.

Three sucrose transporter genes (*SlSUT1*, *SlSUT2*, and *SlSUT4*), the only three members in SUT family in tomato (Reuscher et al., 2014), were analyzed. Among them, *SlSUT1* was dominantly expressed in source leaves but extremely low in reproductive fruit (Figure 2A), while *SlSUT2* and *SlSUT4* were constitutively expressed in both vegetative and different fruit developmental stages (Figures 2B,C). Thus, only the expression of SlSUT2 and SlSUT4 was further investigated in response to elevated CWIN activity in relation to fruit development. The analyses revealed no significant changes of *SlSUT2* and *SlSUT4* mRNA levels in response to elevated CWIN activity during tomato fruit development (Figures 2D,E).

### Elevated CWIN Activity Enhanced the Expression of *SlHT2* and *SlSWEET12c* in Early Tomato Fruit Development

**Figure 3:**
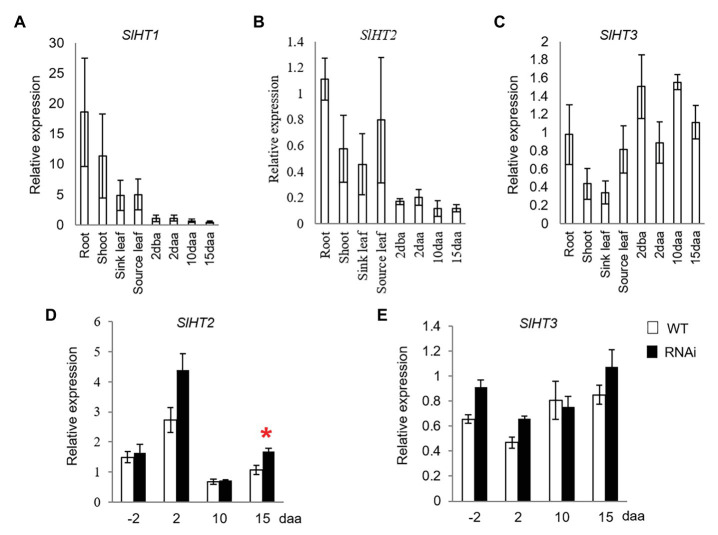
Quantitative real-time-PCR analysis of the expression profiles of hexose transporter (HT) genes. **(A–C)** are expression profiles of HTs in different tissues of tomato plants. **(A)** *SlHT1*. **(B)** *SlHT2*. **(C)** *SlHT3*; **(D)** and **(E)** are expression profiles of HTs in response to elevated CWIN activity in *SlINVINH1*-RNAi as compared to wild type (WT) plants. **(D)** *SlHT2*. **(E)** *SlHT3*. Tissues examined were vegetative and reproductive tissues. Vegetative tissues included root and shoots from 2-week old seedlings, sink, and source leaves from similar raised plants. Reproductive tissues included 2 dba ovaries and 2, 10, and 15 daa fruits. *SlCAC* and *SlSAND* were used as reference genes. Each value is the mean ± SE of three biological replicates. Student *t*-test was applied in comparing *SlINVINH1*-RNAi and WT plants in each individual stage. ^*^Indicates significance at *p* < 0.05.

*SlHT1–3* are the three main contributors for hexose accumulation in tomato fruit (McCurdy et al., 2010). While *SlHT1* was expressed in vegetative tissues, its transcript was only weakly expressed in 2 dba ovaries and developing fruits from 2 to 15 daa (Figure 3A). Expression of *SlHT2* was high in vegetative tissues and 2 dba ovaries, as well as 2, 10, and 15 daa fruits (Figure 3B). Transcript level of *SlHT3* was relatively higher in ovaries and fruits than in vegetative tissues (Figure 3C). The expression level of *SlHT2* significantly increased only in 15 daa fruit of *SlINVINH1*-RNAi (CWIN activity-elevated) when compared to WT plants (Figure 3D), with no response observed for *SlHT3* across all stages examined (Figure 3E).

SWEET transporter family has been previously examined in tomato. There are a total of 29 *SlSWEET* genes as reported by Feng et al. (2015), and 31 *SlSWEET* genes as reported by Ho et al. (2019). SWEET transporters contain seven trans-membranne domains. However, there are two SlSWEET genes containing only six and five TM domains among the 31 SWEETs, respectively (Supplementary Table S6), which are deemed to be non-functional. Hence, on these grounds, two *SlSWWET* genes, *SlSWEET17* and *SlSWEET18*, were excluded from our analyses. Phylogenetic tree of 30 *SlSWEET* genes were clustered with 17 *Arabidopsis SWEET* genes to find closet orthologs in tomato (Supplementary Figure S2). The *SWEET* genes were divided into four clades. Members in clade I, encode transporters such as AtSWEET1a and AtSWEET1b that have been shown to facilitate glucose transport. Members from clade III, such as AtSWEET11 and AtSWEET12 facilitate sucrose efflux (Chen et al., 2012). Therefore, four putative glucose-transporting SWEETs belong to clade I (*SlSWEET1a*, *1b*, *1c*, and *1d*) and three sucrose-transporting SWEETs from clade III (*SlSWEET12a*, *12b*, and *12c*) were selected for further expression analyses (Supplementary Figure S2). Given *SlSWEET1c*, *SlSWEEET1d*, and *SlSWEEET12a* were not expressed during reproductive stages based on semi-RT-PCR analyses (Supplementary Figure S3), only expression of *SlSWEET1a*, *1b*, *12b*, and *12c* was further analyzed during tomato fruit development.

**Figure 4:**
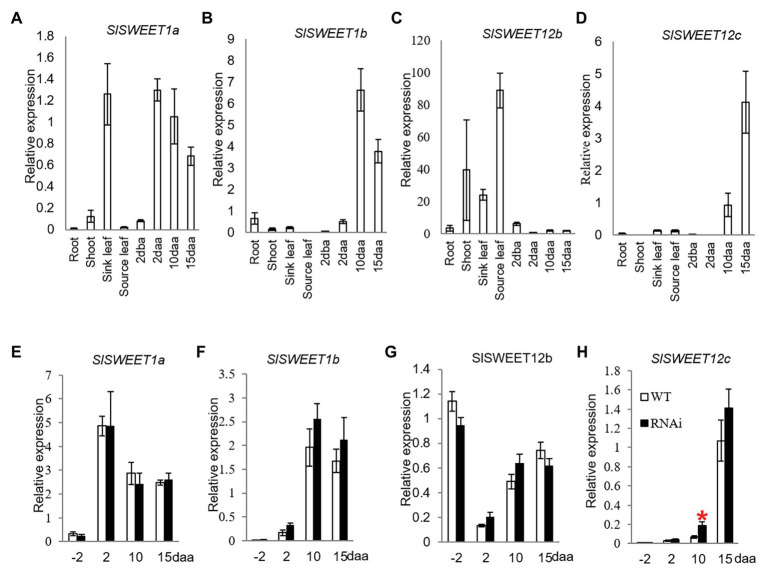
Quantitative real-time-PCR analysis of the expression profiles of sugars will eventually be exported transporter (SWEET) genes. **(A–D)** are expression profiles of SWEETs in different tissues of tomato plants. **(A)** *SlSWEET1a*. **(B)** *SlSWEET1b*. **(C)** *SlSWEET12b*. **(D)** *SlSWEET12c*. **(D–G)** are expression profiles of SWEETs in response to elevated CWIN activity in *SlINVINH1*-RNAi as compared to WT plants. **(E)** *SlSWEET1a*. **(F)** *SlSWEET1b*. **(G)** *SlSWEET12b*. **(H)** *SlSWEET12c*. Tissues examined were vegetative and reproductive tissues. Tissues included 2 dba ovaries and 2, 10, and 15 daa fruits. *SlCAC* and *SlSAND* were used as reference genes. Each value is the mean ± SE of three biological replicates. Student *t*-test was applied in comparing *SlINVINH1*-RNAi and WT plants in each individual stage. ^*^Indicates significance at *p* < 0.05.

Until now, only SlSWEET1a has been experimentally researched among the *SlSWEET* genes in tomato. Here, SlSWEET1a has been shown to be expressed in young leaves, involved in glucose efflux during phloem unloading (Ho et al., 2019); an observation verified in our study (Figure 4A). We further found that the *SlSWEET1a* was abundantly expressed in 2, 10, and 15 daa fruits, with expression levels increased 16-times in 2 daa fruitlets as compared to 2 dba ovaries (Figure 4A). Interestingly, *SlSWEET1b* was only highly expressed in 10 and 15 daa fruits but weakly in 2 daa fruit (Figure 4B). For the three potential *SlSWEET* genes encoding sucrose-transporting from clade III, expression levels of *SlSWEET12b* was abundant in vegetative tissues, especially in source leaves, but low in reproductive tissues (Figure 4C). Similar to the expression pattern of *SlSWEET1b*, the transcripts level of *SlSWEET12c* was highly expressed in 10 and 15 daa fruits (Figure 4D).

Among the *SlSWEET* genes investigated, *SlSWEET1a*, *1b*, and *12b* genes did not show any response in their mRNA levels to elevation of CWIN activity in tomato ovaries and developing fruit across all stages examined in the transgenic plants in comparison to the WT (Figures 4E–G). However, the transcript level of *SlSWEET12c* was significantly increased by 2-fold in 10 daa fruit in response to elevated CWIN activity (Figure 4H).

## Discussion

### CWIN Activity May Be Regulated by Two Inhibitors and One Defective CWIN at the Ovary Stage

Our previous studies have shown that CWIN activity is regulated by *SlINVINH1* at the posttranslational level (Jin et al., 2009). Comparing with fruits, tight regulation of CWIN activity is needed at the ovary stage, probably through interaction with SldeCWIN1 and SlINVINH1, to ensure that the ovaries are well-protected and ready for fruit set upon fertilization (Palmer et al., 2015). Indeed, our transcriptomic analyses revealed that ovaries are more sensitive to elevated CWIN activity as compared to fruitlets (Ru et al., 2017).

In this study, we identified one more inhibitor-*SlINVINH2*, which is specifically expressed at ovary stage. It has been reported that invertase activity could be modulated by INVINHs and defective invertases at the posttranslational level (Jin et al., 2009; Le Roy et al., 2013; Lin et al., 2013). The presence of two INVINHs, *SlINVINH1* and the newly-identified *SlINVINH2*, may act in repressing CWIN activity through the action of SldeCWIN1 at ovary stages to prevent pre-mature cell division in the ovaries prior to fertilization, whereas the near absence of *SldeCWIN1* and *SlINVINH2* expression in 2, 5, and 10 daa fruits could alleviate the inhibition, leading to an increase in CWIN activity to stimulate cell division of the newly formed fruitlets following fertilization. Further experiments are required to determine whether and how SldeCWIN1 and two INHs interact with each other and with CWIN to keep the CWIN activity low prior to fertilization.

### Enhanced Expression of *SlHT2* Could Facilitate Hexose Import Into Fruit Parenchyma Cells

Hexose produced by CWIN in the apoplasm has been shown to be transported into cells by SlHTs. SlHT3 is the main contributor to hexose accumulation in tomato fruit at the middle phase of development (Dibley et al., 2005; McCurdy et al., 2010). Interestingly, it is the expression level of *SlHT2*, but not *SlHT3*, that was increased in response to elevated CWIN activity in 15 daa fruits of the CWIN-elevated transgenic plants (Figure 3D). Fruits at this stage onward are at the onset of rapid hexose accumulation in tomato fruit with sucrose being unloaded apoplasmically from the phloem in the fruit pericarp (Ruan and Patrick, 1995). Extra hexoses released from elevated CWIN activity in 15 daa fruits of the transgenic plants may be taken up by SlHT2 but not SlHT3.

Phloem unloading of sucrose also follows an aoplasmic pathway in the ovary walls and placenta connecting to ovules in 2 dba ovaries (Palmer et al., 2015). However, in contrast to that in 15 daa fruit, no HTs was responsive to elevated CWIN activity in ovaries (Figures 3D,E), indicating that HTs are not sensitive to alteration of CWIN activity or that high expression of HTs is not required at this stage. To this end, it is worth noting that ovaries do not undergo rapid cell division and hexose accumulation, partly owing to low CWIN activity (Palmer et al., 2015) possible through the triple inhibition imposed by SldeCWIN1, SlINVINH1, and SlINVINH2 as hypothesized above. The low CWIN activity and weak expression of *HTs* in the ovaries are also consistent with the fact that only tiny amount of sugar is required to maintain ovary development (Palmer et al., 2015; Ru et al., 2017).

### Enhanced Expression of *SlSWEET12c* Could Facilitated Sucrose Efflux From Phloem to Support Seed Filling

SWEETs are a new class sugar transporter identified over the last 5 years or so (Chen et al., 2015a,b). SWEET may facilitate sucrose or hexose efflux across membranes. Thus, apart from SlHTs, some SWEETs may also facilitate hexose transport. In this context, ZmSWEET4 has been reported to act as a hexose transporter downstream of a CWIN in maize since the expression levels of ZmSWEET4 were suppressed in the CWIN mutant (Sosso et al., 2015). In tomato, SlSWEET1a is localized to the plasma membranes of phloem parenchyma cells of immature leaves to take up glucose from the apoplasm of the phloem likely derived from the CWIN-mediated hydrolysis of unloaded sucrose (Ho et al., 2019). From our phytogenic analysis, SlSWEET1b is another putative hexose-transporting SWEET which might transport hexose into the cytoplasm of sink cells after phloem unloaded sucrose is degraded by CWIN. Expression levels of *SlSWEET1a* and *SlSWEET1b* were not changed in CWIN-elevated fruits (Figures 4E,F). Expression of *SlSWEET1a* showed an increased trend as fruit develops in tomato (Ho et al., 2019). However, this was not the case in our study. Indeed, its expression level decreased in fruit from 2 to 15 daa (Figure 4A). This result indicates that *SlSWEET1a* expression may be cultivar-dependent. Nevertheless, SlSWEET1a seems to act as a glucose effluxer in both vegetative and reproductive organs, such as developing leaves and fruits.

In developing tomato fruit, phloem unloading of sucrose occurs apoplasmically in the sites of placenta connecting to seeds (Jin et al., 2009; Palmer et al., 2015). Here, sucrose must move out of the phloem likely facilitated by clade III SWEET transporters. For instance, the AtSWEET12 facilitates sucrose efflux from phloem of seed coat to embryo in *Arabidopsis* seeds (Chen et al., 2015b). In tomato, the orthologs of AtSWEET12, SlSWEET12b, and SlSWEET12c might play a similar role in facilitating sucrose efflux down a sucrose concentration gradient out of the phloem into seeds of 10 daa fruit. High expression of *CWIN* in this region (Jin et al., 2009; Palmer et al., 2015) could create a steep sucrose gradient between sieve element/companion cell complex and the adjacent apoplasm of the seeds by degrading sucrose into hexose (Patrick, 1997; Ruan, 2014). *SlSWEET12c* is highly expressed in tomato seeds (Supplementary Figure S4) and exhibited an increased expression in response to elevated CWIN activity at 10 daa fruit (Figure 4H). We thus speculate that SlSWEET12c may facilitate sucrose unloading from the phloem down a concentration gradient maintained by CWIN to support seed filling.

**Figure 5:**
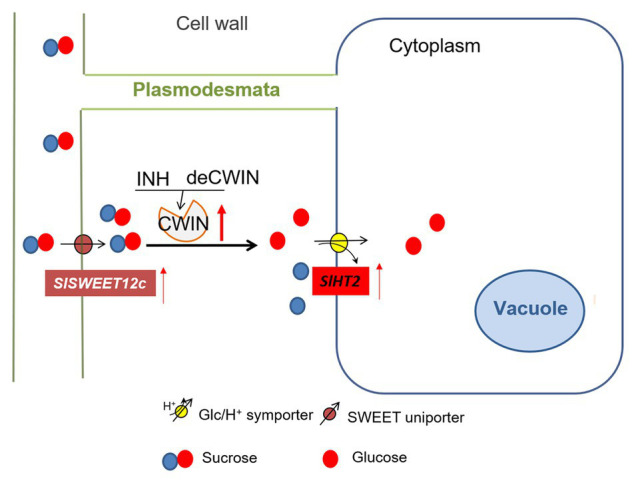
A model of sugar transporters in response to elevated CWIN activity during tomato fruit development. CWIN degarades sucrose (Suc) into glucose (Glc) and fructose. CWIN activity could be tightly regulated by SlINVINHs and/or SldeCWIN1. Suc efflux may be facilitated by Suc-transporting SlSWEETs to the apoplasmic space. Elevated CWIN activity enhanced the expression of *SlSWEET12c*, which may unload extra Suc into cell wall space. Extra Glu produced through elevated CWIN activity is taken up by SlHT2 into the cytosol.

In summary, our results indicate that CWIN activity appears to be tightly controlled by multiple regulators at the ovary stage. Among the sugar transporters investigated, only SWEET12c and SlHT2 were selectively upregulated by elevated CWIN activity during tomato fruit development (Figure 5). The nature of this CWIN-mediated upregulation of these specific sugar transporters is yet to be determined. However, our recent study demonstrates that CWIN positively regulates ovule development through sugar signaling that is potentially perceived by a cohort of HTs and clades I and II SWEETs (Liao et al., 2020). Experiments are underway to test these possibilities along with the functional implications of this coupling between sucrose catabolism and sugar transport in fruit development.

## Data Availability Statement

All datasets presented in this study are included in the article/Supplementary Material.

## Author Contributions

Y-LR conceived the project. LR and Y-LR designed the experiments. LR and YH conducted the experiments. LR, Y-LR, and JP analyzed the data. LR, Y-LR, JP, YH, and ZZ wrote the manuscripts. All authors contributed to the article and approved the submitted version.

### Conflict of Interest

The authors declare that the research was conducted in the absence of any commercial or financial relationships that could be construed as a potential conflict of interest.
