# Supplementary material for: Integrating Sugar Metabolism With Transport: Elevation of Endogenous Cell Wall Invertase Activity Up-Regulates SlHT2 and SlSWEET12c Expression for Early Fruit Development in Tomato
Source: Front Genet. 2020 Oct 19;11:592596. doi: 10.3389/fgene.2020.592596 (PMC7604364; doi:10.3389/fgene.2020.592596)
Supplement: Supplementary file 1 [file Data_Sheet_1.docx]

Supplementary Material

***
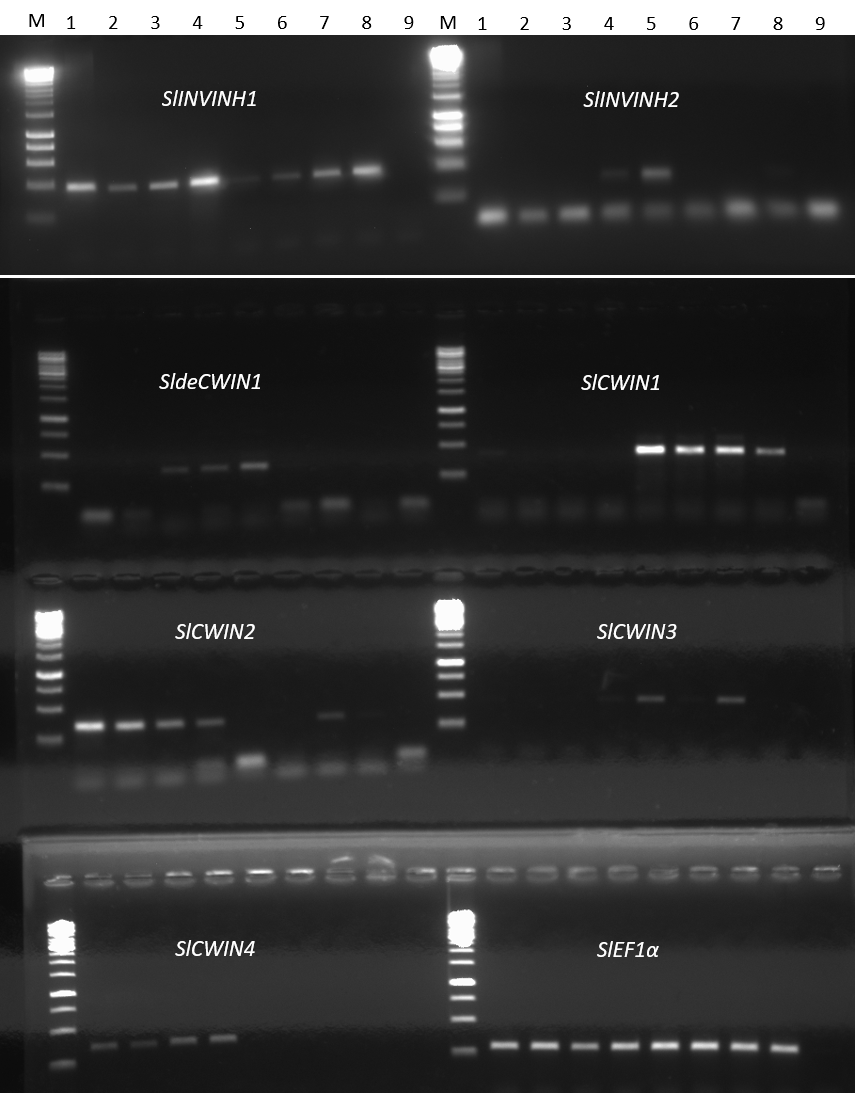
***

Supplementary Figure S1: Original gel images of CWIN and its potential regulators in Figure 1A.

1, roots. 2, shoots from 2-week old seedlings, 3, sink leaves. 4, source leaves. 5, 2 days before anthesis (2 dba) ovaries. 6, two days after anthesis (daa) fruits (2daa). 6, two days after anthesis (daa) fruits (2 daa). 7, five days after anthesis (daa) fruits (5 daa). 8, ten days after anthesis (daa) fruits (10 daa). SlEF1α was used as reference gene.


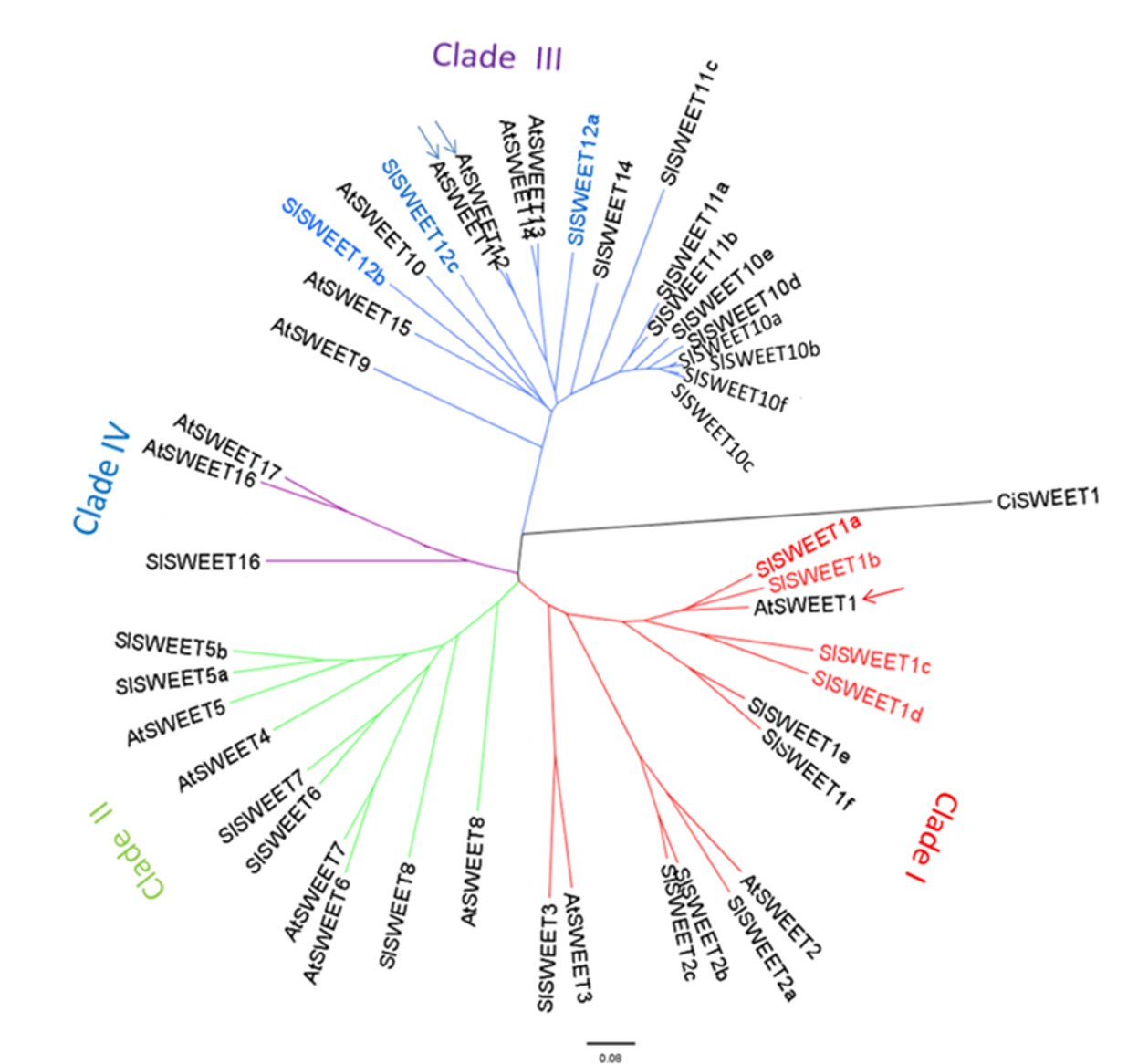


Supplementary Figure S2: Phylogenetic tree of SWEET transporters.

The phylogenetic tree was generated with MEGA 5.10 using the neighbour-joining method following multiple sequences alignments with ClustalW. SWEETs genes fall into four clades as showed in different colours. Putative glucose-transporting SWEETs were highlighted in red and putative sucrose-transporting were highlighted in blue for tomato SWEETs. CiSWEET1 was used as an outlier. Species abbreviation: At, *Arabidopsis thaliana*; Ci: *Ciona intestinalis;* Sl, *Solanum lycopersicum*. For Accession numbers, CiSWEET1 (AK114161). Accession numbers for SlSWEETs and AtSWEETs were listed in Supplemental Table 6.


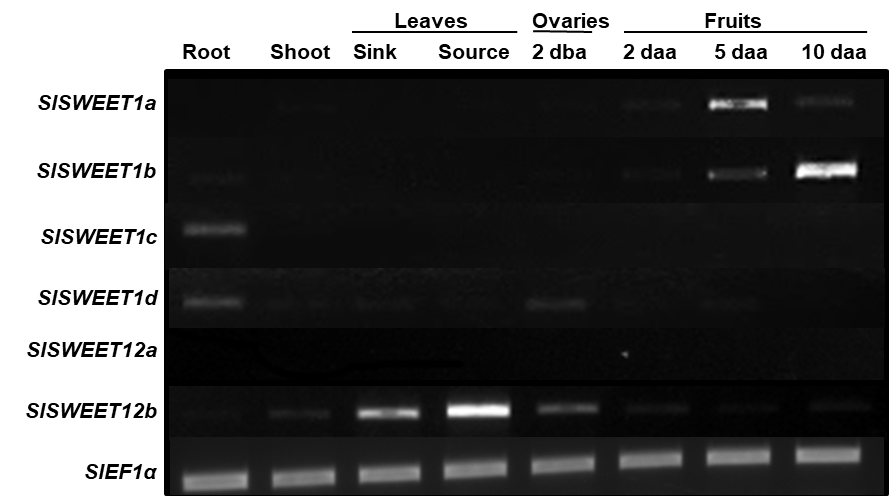


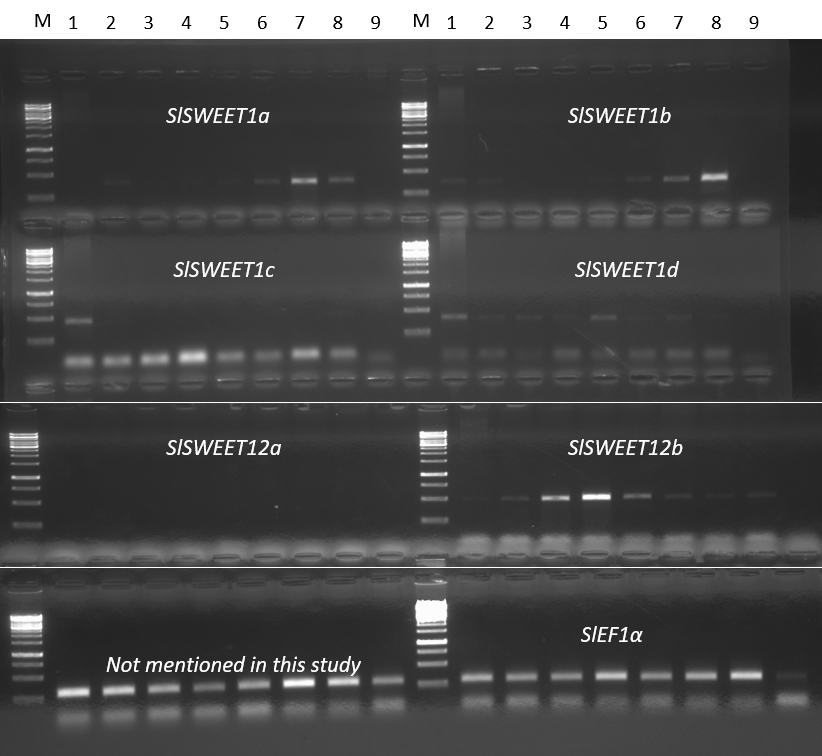


Supplementary Figure S3: Semi-qRT-PCR analysis of the expression profiles of SWEET genes.

Expression profile of SWEETs (*SlSWEET1a, 1b, 1c, 1d, 12a, and 12b*). Vegetative tissues included roots and shoots from 2-week old seedlings, sink and source leaves from the same grown up plants. Reproductive tissues included 2 days before anthesis (2 dba) ovaries and 2, 5, 10 days after anthesis (daa) fruits. *SlEF1α* was used as reference gene. Upper panel showed the cropped images, lower panel showed the original gels of genes in the upper panel (M: 1000bp marker; 1-8 represent the same tissues as in the upper panel, 9 is negative control).


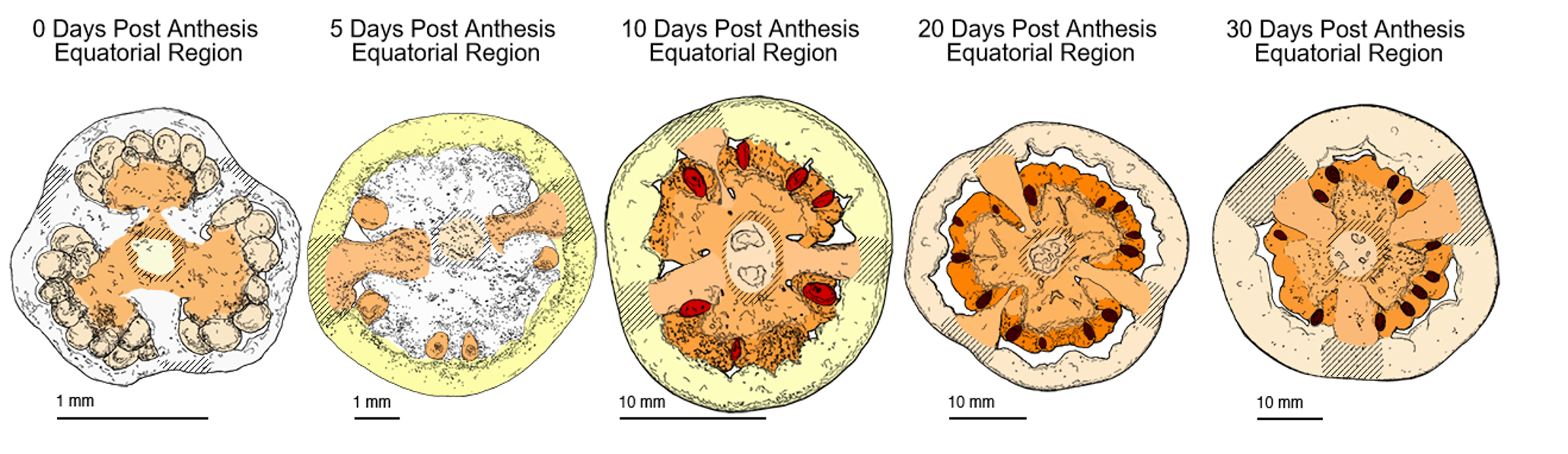


Supplementary Figure S4: Expression profiles of SlSWEET12c inside tomato ovary or fruits.

Data were acquired from http://tea.sgn.cornell.edu/.

Supplementary Table S1: Primer sets for semi-qRT-PCR.

| **Genes** | **Sequence(5'-3')** | | **Predicted size(bp)** |
| --- | --- | --- | --- |
| *SldeCWIN1* | Forward | TGCTTATGCCAGGATCAATAC | 324 |
|  | Reverse | CATAATCACTAGAAGGCAAGC |  |
| *SlCWIN1* | Forward | ACAAGCCCTCATTTGCTGGA | 405 |
|  | Reverse | CCATACACACACTTTGCCTTC |  |
| *SlCWIN2* | Forward | GATGCCTCAAGGTCAAGCCT | 333 |
|  | Reverse | TACTATGGTTTCTTTGTGACGTGGC |  |
| *SlCWIN3* | Forward | CTTGGGCCATTTGGTCTTGC | 446 |
|  | Reverse | CCCCTTTTACCATAGTTCCTTTCTCCT |  |
| *SlCWIN4* | Forward | TGCCTCAAGATCAACCCTCG | 365 |
|  | Reverse | GAGCAATCAAATACGTCACCACAAC |  |
| *SlINVINH1* | Forward | CCAGCAAGTATGCCAGAAGC | 375 |
|  | Reverse | GGGGATACACACATAACATTTGAGG |  |
| *SlINVINH2* | Forward | ACAAAAGAAGTGAAAAAGCAGGAGA | 265 |
|  | Reverse | GCATCTCCAGAAGTACCTACCA |  |
| *SlSWEET1a* | Forward | TTGCATTTGGCCTTCTTGGC | 373 |
|  | Reverse | GGTTTATTTGAGCAGGCAGCA |  |
| *SlSWEET1b* | Forward | TAGTGGCTTTGCTGCTGCTAT | 334 |
|  | Reverse | TGCATCACCATTTTGGGTGTT |  |
| *SlSWEET1c* | Forward | ACATGCCCTTCTTCTTGTCACT | 442 |
|  | Reverse | GCAATTACAACAAACACCTAGCAA |  |
| *SlSWEET1d* | Forward | CGTGGCCTTAGTTTCCCTGT | 390 |
|  | Reverse | CACGCGTCTTCGTTGTAAGC |  |
| *SlSWEET12a* | Forward | TGGATGGATTTGCATGGCTT | 312 |
|  | Reverse | GGCTGCTCTTGATCCACCTT |  |
| *SlSWEET12b* | Forward | ACCGTAGCAGTGGGTGTTTT | 467 |
|  | Reverse | CATGCAGCACACACCACAAG |  |
| *SlLeEF1α* | Forward | TGCATTGCTTGCTTTCACCC | 279 |
|  | Reverse | GGCCTCTTGGGCTCGTTAAT |  |

Supplementary Table S2: Candidate reference genes for qRT-PCR

| **Symbol** | **Name** | **Tomato accession number/Locus ID** | **Function/ annotation** |
| --- | --- | --- | --- |
| SlACTIN | Beta actin | XM_004236699 | Cytoskeletal structural protein |
| SlGAPDH | Glyceraldehyede-3-phosphate dehydrogenase | Solyc05g014470.2 | Oxidoreductase in glycolysis and gluconeogenesis |
| SlEF1a | Elongation factor 1 alpha | Solyc06g009970.2 | Translation elongation |
| SlCAC | Clathrin adaptor complexes medium subunit | Solyc08g006960.2 | Endocytic pathway |
| SlExpressed | Expressed sequence | Solyc07g025390.2 | Peptidyl-prolyl cis-trans isomerase cyclophilin-type |
| SlTIP41 | - | Solyc01g107420.2 | TIP41-like family protein |
| SlSAND | Sand family protein | Solyc03g115810.2 | Vacuolar fusion protein mon1 |

Supplementary Table S3: Primer sets for qRT-PCR

| **Genes** | **Sequence(5'-3')** | | **Predicted size(bp)** |
| --- | --- | --- | --- |
| *SldeCWIN1* | Forward | AGTATGAGAGGATGGGCTGG | 191 |
|  | Reverse | TCTGCCTGTGTAGCATTGAC |  |
| *SlCWIN1* | Forward | TGAGACTCTGAATGCTTGGAG | 200 |
|  | Reverse | CCATACACACACTTTGCCTTC |  |
| *SlINVINH1* | Forward | GCATTCTAATCCTCCTCAAGC | 173 |
|  | Reverse | CTTCACATTCTTGTGCATCACC |  |
| *SlHT1* | Forward | CGATGATCGAACGTGGTAAC | 146 |
|  | Reverse | CAACAAGTTCCTCCAGGGAT |  |
| *SlHT2* | Forward | TCAACTACGGAACAGCCAAG | 199 |
|  | Reverse | TCAGGTTCAATGTTGTCGGT |  |
| *SlHT3* | Forward | TGCGATAGCACAGTCTTTCC- | 121 |
|  | Reverse | CTTCGTTTCAGGCAAGAACA |  |
| *SlSUT1* | Forward | TTCCATAGCTGCTGGTGTTC | 128 |
|  | Reverse | TACCAGAAATGGGTCCACAA |  |
| *SlSUT2* | Forward | CCTACAGCGTCCCTTTCTCT | 116 |
|  | Reverse | GGATACAACCATCTGAGGTACAA |  |
| *SlSUT4* | Forward | TCTCCGCTGATATTGGATGG | 97 |
|  | Reverse | GCAACATCGAGAAGCCAAAA |  |
| *SlSWEET1a* | Forward | CGTTGTTTGTGTTCTTATGTGG | 209 |
|  | Reverse | AGATTGCTTCTCCTCTTGGTG |  |
| *SlSWEET1b* | Forward | GTCCTTGTTTGTCTTTCTATGTGG | 184 |
|  | Reverse | CTCTACTCTTCCATCTTCCTCAC |  |
|  |  |  | Table continues on next page |
| **Supplementary Table S3**: continued from previous page | | | |
| *SlSWEET12b* | Forward | TCATTCTCTACGCATCCAAGG | 162 |
|  | Reverse | CCACTGCTACGGTTACACAA |  |
| *SlSWEET12c* | Forward | TGACATGAAAGCGGTGGTGG | 234 |
|  | Reverse | TACCTTTGGCTATTAGGGGGC |  |
| *SlACTIN* | Forward | GGAATAGCATAAGATGGCAGACG | 159 |
|  | Reverse | ATACCCACCATCACACCAGTAT |  |
| *SlGAPDH* | Forward | GGCTGCAATCAAGGAGGAA | 207 |
|  | Reverse | AAATCAATCACACGGGAACTG |  |
| *SlEFα1* | Forward | TACTGGTGGTTTTGAAGCTG | 166 |
|  | Reverse | AACTTCCTTCACGATTTCATCATA |  |
| *SlTIP41* | Forward | ATGGAGTTTTTGAGTCTTCTGC | 235 |
|  | Reverse | GCTGCGTTTCTGGCTTAGG |  |
| *SlSAND* | Reverse | TTGCTTGGAGGAACAGACG | 164 |
|  | Forward | GCAAACAGAACCCCTGAATC |  |
| *SlCAC* | Reverse | CCTCCGTTGTGATGTAACTGG | 173 |
|  | Forward | ATTGGTGGAAAGTAACATCATCG |  |
| *SlExpressed* | Reverse | GCTAAGAACGCTGGACCTAATG | 183 |
|  | Forward | TGGGTGTGCCTTTCTGAATG |  |

Supplementary Table S4：Input data for Genorm in ranking reference genes in different tissues of tomato plants.

| **Tissues** | ***SlTIP41*** | ***SlCAC*** | ***SlGAPDH*** | ***SlEF1α*** | ***SlEXPRESSED*** | ***SlACTIN*** | ***SlSAND*** |
| --- | --- | --- | --- | --- | --- | --- | --- |
| Shoot | 1.00 | 0.82 | 0.37 | 0.89 | 0.21 | 0.53 | 0.75 |
| Root | 0.89 | 0.59 | 0.29 | 0.29 | 0.14 | 0.19 | 0.43 |
| Source leaves | 0.72 | 0.51 | 0.11 | 0.24 | 0.11 | 0.29 | 0.45 |
| Sink leaves | 0.64 | 0.61 | 0.29 | 0.34 | 0.20 | 0.43 | 0.53 |
| 2d ba ovary | 0.79 | 0.97 | 0.22 | 0.49 | 0.34 | 0.17 | 1.00 |
| 2 daa fruit | 0.95 | 0.89 | 0.48 | 1.00 | 0.43 | 0.31 | 0.72 |
| 10 daa fruit | 0.76 | 1.00 | 0.55 | 0.79 | 1.00 | 1.00 | 0.82 |
| 15 daa fruit | 0.74 | 0.90 | 1.00 | 0.61 | 0.33 | 0.36 | 0.92 |
| The Ct values were transformed to quantities by using the comparative Ct method, and then the highest relative quantities for each gene are set to 1. | | | | | | | |

Supplementary Table S5： Comprehensive ranking of reference genes by Genorm

| **Rank** | **Reference genes** |
| --- | --- |
| *1* | *SlCAC/SlSAND* |
| *2* | *SlTIP41* |
| *3* | *SlEF1α* |
| *4* | *SlEXPRESSED* |
| *5* | *SlGAPDH* |
| *6* | *SlACTIN* |
| The rank is from the most stable (1) to unstable (6). Group 1 included roots and shoots from 2-week old seedlings, sink leaves, source leaves and 2 days before anthesis (2 dba) ovaries and 2, 10, 15 days after anthesis (daa) fruits. | |

Supplementary Table S6： Nomenclature of invertase, invertase inhibitors and related sugar transporters in tomato.

| *CWINs SldeCWIN1 Solyc03g121680*  *SlCWIN1 Solyc09g010080*  *SlCWIN2 Solyc10g083290*  *SlCWIN3 Solyc09g010090*  *SlCWIN4 Solyc10g083300* | SldeCWIN1 | Solyc03g121680 | *SWEETs* | SlSWEET2b | Solyc03g005880 |
| --- | --- | --- | --- | --- | --- |
|  | SlCWIN1 | Solyc09g010080 |  | SlSWEET2c | Solyc02g071520 |
|  | SlCWIN2 | Solyc10g083290 |  | SlSWEET3 | Solyc03g007360 |
|  | SlCWIN3 | Solyc09g010090 |  | SlSWEET5a | Solyc03g114200 |
|  | SlCWIN4 | Solyc10g083300 |  | SlSWEET5b | Solyc06g071440 |
|  |  |  |  | SlSWEET6 | Solyc08g082770 |
| *INHs* | SlINVINH1 | Solyc12g099200 |  | SlSWEET7 | Solyc12g055870 |
|  | SlINVINH2 | Solyc12g099210 |  | SlSWEET8 | Solyc02g086920 |
|  | SlINVINH3 | Solyc12g099190 |  | SlSWEET10a | Solyc03g097570 |
|  |  |  |  | SlSWEET10b | Solyc03g097580 |
| *SUTs* | SlSUT1 | Solyc11g017010.1 |  | SlSWEET10c | Solyc03g097590 |
|  | SlSUT2 | Solyc05g007190.2 |  | SlSWEET10d | Solyc03g097600 |
|  | SlSUT4 | Solyc04g076960.2 |  | SlSWEET10e | Solyc03g097610 |
|  |  |  |  | SlSWEET10f | Solyc03g097870 |
| *HTs* | SlHT1 | Solyc02g079220.2 |  | SlSWEET11a | Solyc06g072620 |
|  | SlHT2 | Solyc09g075820.2 |  | SlSWEET11b | Solyc06g072630 |
|  | SlHT3 | Solyc07g006970.2 |  | SlSWEET11c | Solyc06g072640 |
|  |  |  |  | SlSWEET12a | Solyc03g097620 |
| *SWEETs* | SlSWEET1a | Solyc04g064610 |  | SlSWEET12b | Solyc05g024260 |
|  | SlSWEET1b | Solyc04g064620 |  | SlSWEET12c | Solyc09g074530 |
|  | SlSWEET1c | Solyc04g064630 |  | SlSWEET14 | Solyc03g097560 |
|  | SlSWEET1d | Solyc04g064640 |  | SlSWEET16 | Solyc01g099880 |
|  | SlSWEET1e | Solyc06g060580 |  | SlSWEET17 | Solyc01g09987 |
|  | SlSWEET1f | Solyc06g060590 |  | SlSWEET18 | Solyc11g028270 |
|  | SlSWEET2a | Solyc07g062120 |  |  |  |
| SlSWEET1-16 contains 7 transmembrane domains, SlSWEET17 contains 6 transmembrane domains, SlSWEET18 contains 5 transmembrane. TMHMM Server v.2.0 was used to predict transmembrane domains (<http://www.cbs.dtu.dk/services/TMHMM/>). | | | | | |
